# Structural Monitoring Without a Budget—Laboratory Results and Field Report on the Use of Low-Cost Acceleration Sensors

**DOI:** 10.3390/s25154543

**Published:** 2025-07-22

**Authors:** Sven Giermann, Thomas Willemsen, Jörg Blankenbach

**Affiliations:** 1 Hochschule Neubrandenburg Landscape Sciences and Geomatics, Brodaer Straße 2, 17033 Neubrandenburg, Germany; 2 Geodetic Institute and Chair for Computing in Civil Engineering & GIS, RWTH Aachen University, Mies-van-der-Rohe-Str. 1, 52074 Aachen, Germany

**Keywords:** low-cost sensors, SHM, inclination sensors, MEMS accelerometers, structural monitoring

## Abstract

Authorities responsible for critical infrastructure, particularly bridges, face significant challenges. Many bridges, constructed in the 1960s and 1970s, are now approaching or have surpassed their intended service life. A report from the German Federal Ministry for Digital and Transport (BMVI) indicates that about 12% of the 40,000 federal trunk road bridges in Germany are in “inadequate or unsatisfactory” condition. Similar issues are observed in other countries worldwide. Economic constraints prevent ad hoc replacements, necessitating continued operation with frequent and costly inspections. This situation creates an urgent need for cost-effective, permanent monitoring solutions. This study explores the potential use of low-cost acceleration sensors for monitoring infrastructure structures. Inclination is calculated from the acceleration data of the sensor, using gravitational acceleration as a reference point. Long-term changes in inclination may indicate a change in the geometry of the structure, thereby triggering alarm thresholds. It is particularly important to consider specific challenges associated with low measurement accuracy and the susceptibility of sensors to environmental influences in a low-cost setting. The results of laboratory tests allow for an estimation of measurement accuracy and an analysis of the various error characteristics of the sensors. The article outlines the methodology for developing low-cost inclination sensor systems, the laboratory tests conducted, and the evaluation of different measures to enhance sensor accuracy.

## 1. Introduction

The stability and resilience of infrastructure facilities are crucial for the safety and economic viability of a country. In Germany, the USA, and many other countries where significant expansion of transportation infrastructure took place in the 1960s and 1970s, recent reports, along with incidents such as the collapse of the Carola Bridge in Dresden, highlight a concerning state of bridge infrastructure. According to a report by the Federal Ministry of Transport and Digital Infrastructure (BMVI), about 12% of the approximately 40,000 bridges in Germany are in insufficient or inadequate condition [1]. Similarly alarming is the situation in the USA, where the American Society of Civil Engineers (ASCE) reports that around 42% of all bridges are at least 50 years old [2], with 6.8% classified as structurally deficient [3]. These figures underscore the urgency for continuous monitoring and maintenance. In this context, low-cost MEMS acceleration sensors offer a promising way to improve the monitoring of bridges and other critical infrastructures efficiently and economically. 

MEMS (Micro-Electro-Mechanical Systems) acceleration sensors use silicon and other microfabricated materials to convert mechanical movements into electrical signals. The capacitive measurement method measures the movement of a microscopic mass that is attached to springs and suspended between two electrodes. During acceleration, the distance to the electrodes changes, affecting the capacitance, which is converted into an electrical signal. This paper presents studies on the applicability of low-cost acceleration sensors for continuous static structural monitoring. Currently, assessments of a bridge’s condition, including bearings and expansion joints, are typically carried out through visual inspections at regular intervals. Major inspections, which occur every six years, also include measurements of geometry [4]. Visual inspection is particularly subjective, providing only limited quantitative data on bridge elements, and it has been shown to yield very different qualitative information [5,6]. MEMS low-cost sensors offer advantages such as dense network coverage and more detailed data acquisition. They are small and unobtrusive, facilitating strategic placement. These sensors enable continuous long-term monitoring, identify trends, and, when handled properly, help avoid costly repairs.

### 1.1. SHM and Research on the Adaptation of Low-Cost Sensor Technology

Structural Health Monitoring (SHM) is a comprehensive approach that employs various technologies to assess the condition and performance of bridge structures. It includes three main components: data acquisition and transmission using sensors to measure physical quantities such as strain, vibrations, or deformations. Then, data preprocessing serves to clean and prepare data for analysis to increase accuracy. Finally, condition assessment involves damage detection and the deployment of early warning systems for early detection of potential problems that could lead to failures. In the area of data acquisition and sensor technologies, accelerometers and strain gauges play a significant role due to their widespread application, as do laser-based and tachymetric systems. However, other technologies are increasingly being used in the context of SHM: piezosensitive sensors [7,8] measure high-frequency structural vibrations or, in altered configurations, such as semiconductor strain gauges, measure strains or deformations on infrastructure objects. Their measurement principle is based on the fact that the deformation changes the crystal lattice structure in certain materials (e.g., quartz, PZT, PVDF, etc.), leading to a change in charge (piezoelectric effect), capacitance (piezocapacitive effect), or resistance (piezoresistive effect), which can be measured and is proportional to the strain or compression. Triboelectric nanogenerators (TENGs) should also be mentioned. They rely on the principle of electrostatic charging (“friction electricity”) and discharging. TENGs use vibrations on objects, which can be utilised as an alternating voltage and current flow in the TENG through a constant charging and discharging process in the context of SHM. Challenges to the extended application in SHM for bridges include, in addition to their limited energy yield, sensitivity to environmental conditions. However, they, along with piezoelectric sensors, have the potential to operate electrically autonomously under certain circumstances due to their underlying physical measurement principle, which makes them particularly interesting for deployments in remote locations [9].

A research field that is also increasingly gaining focus is the use of fibre optic sensors (FOS) in the context of SHM. They allow for continuous and distributed measurement of parameters such as strain, temperature, or even acoustic signals over long distances. Special technological implementations such as Fibre Bragg Gratings (FBGs) allow for multiplexing of numerous measurement points along a single fibre, facilitating installation and monitoring. However, high costs, sensitive installation, and complex signal processing pose challenges for their application [10,11].

All these sensor technologies belong to passive methods, as they only capture the buildings behaviour but do not interact with the structure. In a second category of technology applications in SHM, active methods are used, where actuators act on the structure, and the object’s response is measured and evaluated in the next step. This includes methods such as stress wave analysis using piezoelectric sensors, the use of ultrasonic waves (see [12]), or electromagnetic impedance analysis (EMIA). Here, piezoelectric sensors are used as actuators (inverse piezoelectric effect) to excite a structure with high-frequency mechanical oscillations in the kilo- or megahertz range. The propagation and dissipation of the waves in the object’s structure correlate with the electrical impedance, which is recorded as a system response in the sensor. The electrical impedance has a real part, which is a measure of the dissipation of mechanical energy in the structure, and an imaginary part, which is a measure of the elasticity of the structure. Changes in these parameters indicate damage to the object’s structure, such as increased vibration dissipation due to cracks [13].

An advanced SHM system, in addition to the sensors, includes wireless transmission technologies. They enable real-time data transmission and avoid issues associated with wired systems, such as difficult installation and maintenance in some cases, or problems related to electromagnetic shielding, as well as higher costs [9]. Data preprocessing methods such as data cleaning, fusion, transformation, reduction, and feature extraction are critical for controlling noise and improving the quality of data used for analysis. In addition to the evaluation of data by specialist personnel, there are systematic methods of damage detection. These methods are typically classified as either model-based or data-driven and assist in the identification of structural anomalies and potential failures. Practical applications of SHM include early warning systems that trigger alarm events based on the static or dynamic responses of a structure when a defined threshold is exceeded. The static response of an object refers to its long-term change, such as the gradual deflection of a steel or concrete beam over the years. The dynamic response of a structure refers to its short-term behaviour concerning excitation by load cases, such as vibrations due to traffic, wind, or thermal loads. These warning systems are not only applied concerning structural ageing processes but also concerning the response of engineering structures to disaster events such as earthquakes, extreme flood waves, or ship collisions with bridge piers. 

Further literature on the topic includes [9,12,14], providing a good overview of current SHM concepts, while [15,16] focus on the sensors used.

In the field of utilising low-cost sensors in SHM, research is predominantly concentrated on the following domains:

- The use of cost-efficient sensors, particularly MEMS acceleration sensors [17,18,19] [20,21], but also other types of sensors such as piezoelectric sensors [22] or single-fibre FOS [23].
- The calibration and compensation of temperature effects of MEMS acceleration sensors [24,25,26].
- The use of open-source platforms such as Arduino or Raspberry Pi while ensuring data quality and security [27,28,29,30].
- The use of wireless data transmission and IoT-connected devices [31,32].
- The synchronisation of different measurement nodes [33].
- The combination of MEMS acceleration sensors with other technologies like GNSS, gyroscopes, or even vision-based approaches, to improve data quality [34,35,36,37,38,39].
- The use of AI-based data evaluation methods [40,41,42].

Note that the attached literature references are to be understood as representative in this area. The objective of the research conducted within the low-cost domain of the SHM is to identify cost-effective solutions that can address the requirement for large-scale monitoring solutions in the context of the aforementioned situation, characterised by the persistent need for viable monitoring systems that ensure the safe operation of ageing bridges. A subfield of this research is the precise capture of gradually occurring geometric change processes or long-term deformations of bridge components. As opposed to the short-term, high-frequency measurements of modal parameters used in dynamic analysis, this is known as static analysis.

### 1.2. Static Analysis Using MEMS Acceleration Sensors

**Figure 1:**
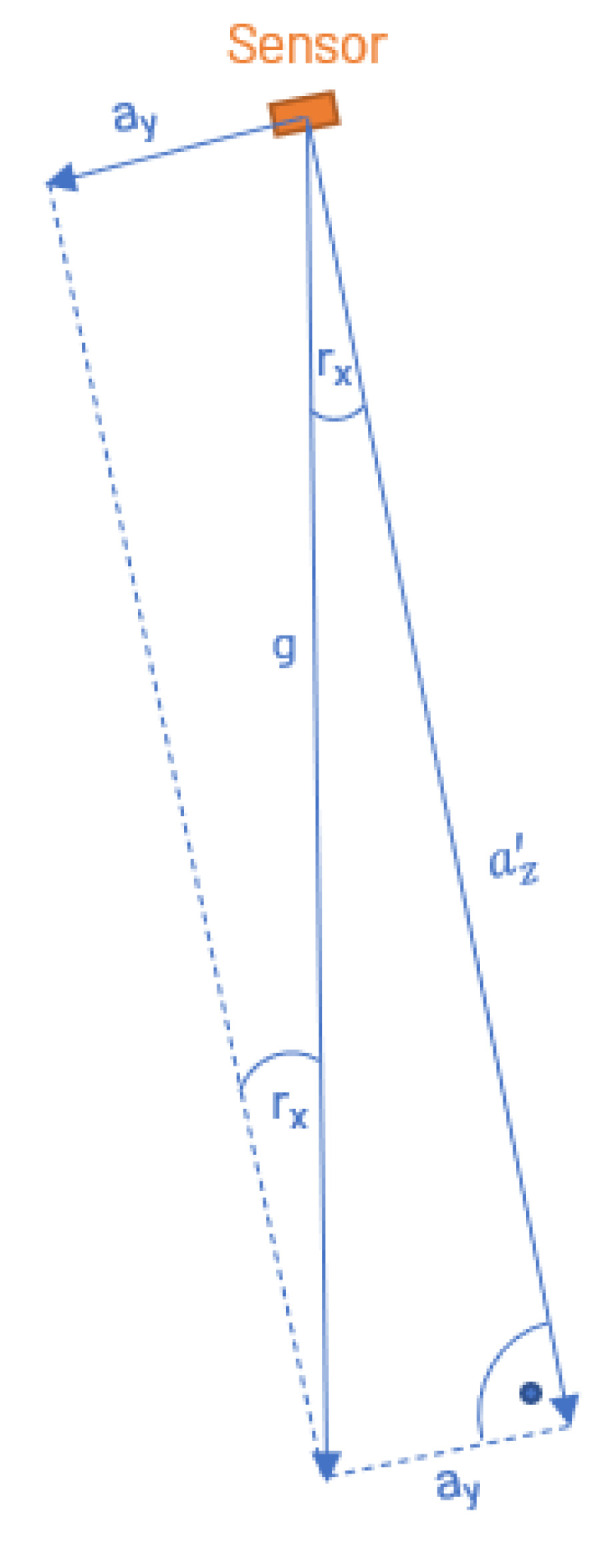
A 3 axes acceleration sensor in relation to g, y-z plane, in case $a_{x}=0$ becomes $a_{z}^{\prime }=a_{z}$, in case $a_{x}\neq 0$ becomes $a_{z}^{\prime }=\sqrt{a_{x}^{2}+a_{z}^{2}}.$

MEMS accelerometers can be used as tilt sensors by measuring the components of gravitational acceleration along their three axes when at rest and determining the inclination relative to the natural vertical from the ratios between these components. Figure 1 schematically depicts the relationship between the inclination of a sensor’s axes and the gravitational force (g) in a two-dimensional plane:

Trigonometric functions provide the mathematical relationship for this. Thus, an inclination around the x-axis is given by

(1) $$tanr_{x}=\frac{a_{y}}{\sqrt{a_{x}^{2}+a_{z}^{2}}}$$

and accordingly,

(2) $$r_{x}=arctan\left(\frac{a_{y}}{\sqrt{a_{x}^{2}+a_{z}^{2}}}\right)$$

Similarly, for an inclination around the y-axis:

(3) $$r_{y}=arctan\left(\frac{a_{x}}{\sqrt{a_{y}^{2}+a_{z}^{2}}}\right)$$

Inclination values determined in this way can then provide information about the displacement at the observed point, based on the structural knowledge:

(4) $$h=d\cdot sinr_{x}$$

**Figure 2:**
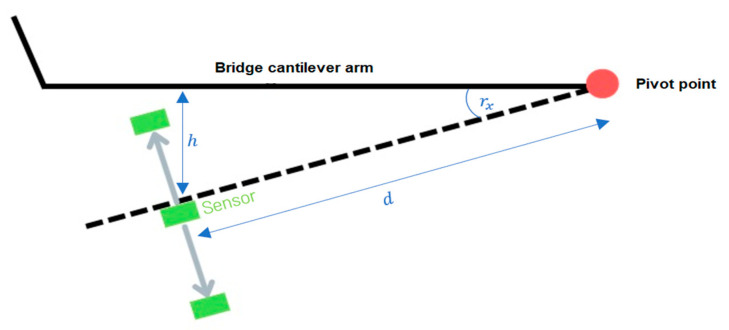
Schematic representation of displacement measurements with an accelerometer.

As schematically indicated in Figure 2, critical lowering of cantilever arms on bridges can be monitored, as per [43], or movements at bridge supports and expansion joints can be checked, as per [44]. This type of monitoring is long term. Requirements regarding maximum permissible values for inclinations or deflections in millimetres are defined as tolerances and vary from one object to another. They are derived, for example, from FEM models of the object, associated calculations, structural assessment reports, empirical baseline monitoring (load testing upon commissioning as baseline), or national and international standards. Permissible movements and deflections of bridge components strongly depend on the type of deformation and the component itself. For example, the roadway of large suspension bridges can be deflected several decimetres under certain circumstances, whereas the deflection of concrete beams on short bridges is in the range of a few millimetres. Changes in inclination are usually in the range of <0.5° [18]. In a specific application case of a monitoring campaign on a box girder bridge, the Benediktus Bridge in Düsseldorf, an alarm threshold of 20 mm is defined for the outer end of the cantilever of the prestressed concrete structure. Within the monitoring concept, a tolerance for detectable movements of 2 mm at the location is specified. Given the requirement for a measurement instrument with a standard deviation five times smaller than the given tolerance [45] to confidently detect the required threshold, an accuracy demand of approximately 0.007° (1σ) of inclination change arises. This is based on the length of the cantilever arm in cross-section of the bridge being 3.24 m, which also corresponds to the distance from the pivot point of the displacement motion to the point of maximum deflection at the outer end of the cantilever $\left(arcsin\frac{\frac{2mm}{5}}{3.24m}≅0.007{}^{\circ}\right)$. Generally, it should be noted in SHM that the focus is not solely on isolated absolute measurements in the context of tolerance limits but also on the nature and context of changes in these values. Therefore, caution is generally advised with long-term increases in deflection maxima or sudden changes in values [46]. Furthermore, it should be noted that although knowledge of the angle of inclination of various points on the object being monitored can provide helpful information about its condition, it cannot replace a comprehensive and holistic condition analysis based on additional, different measurement variables and physical parameters, such as dynamic vibration characteristics or modal parameters.

## 2. System Setup and Preliminary Investigation

A prototype was developed for the studies described in this paper, allowing for estimates of the achievable accuracy of the MEMS module under laboratory conditions, and later demonstrated the feasibility of structural monitoring on a specific object, the Benediktus Bridge in Düsseldorf. The prototype consists of 10 MEMS accelerometers of the type MPU-9250 (refer to the datasheet: https://invensense.tdk.com/wp-content/uploads/2015/02/PS-MPU-9250A-01-v1.1.pdf, accessed on 1 May 2025) which are connected to a small circuit board with an ESP32 microcontroller to store, read, and transmit data.

**Figure 3:**
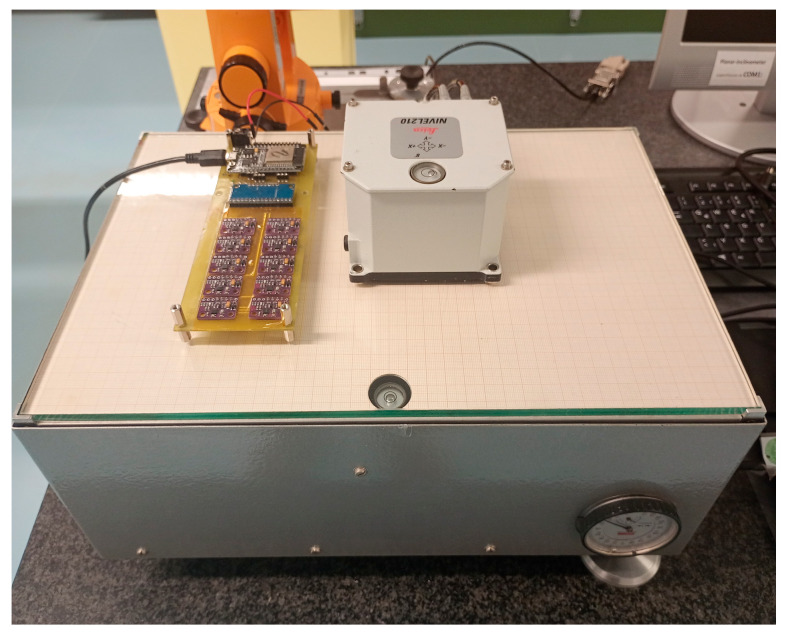
PCB with 10 MEMS accelerometer (MPU-9250) and Nivel210 as reference.

The primary purpose of the large number of sensors in the initial phase was to achieve improved overall accuracy through averaging. Moreover, under laboratory conditions (T = constant = 21 °C), the extent to which accuracy improves with the increased number of sensors used for averaging was determined. Defined inclination angles from −0.15° to +0.15° were set on an inclination test bench, and 17 measurement points were distributed evenly across this range. The setup of the experiment is shown in Figure 3.

These set inclinations were measured by both the sensor module, which includes the 10 MEMS accelerometers, and the reference device Nivel210. The reference device’s accuracy in the measurement range of −0.08° to +0.08° (1*σ*, technical datasheet for Leica Nivel 210 available at https://leica-geosystems.com/de-de/products/total-stations/systems/geotechnical-sensors/leica-nivel210_220, accessed on 1 May 2025) is ≈22 times better than the best accuracy achieved with the MPU-9250 sensors by the authors in the laboratory. For larger inclination angles, the accuracy of the reference device declines but never falls below a factor of 5 compared to the accuracy of the MEMS sensors. The values from the reference device and the MEMS sensors were compared, and the accuracy of the sensor module was derived using the RMSE (root mean square error):

(5) $$RMSE=\sqrt{\sum_{i=1}^{n}\frac{{\left(r_{i}-r_{i,ref}\right)}^{2}}{n}}$$

where

$r_{i}\;$is the measured inclination (average) of the sensor module at inclination position i.

$r_{i,ref}$ is the measured reference value at inclination position i.

Each measurement position was set for approximately 30 s, and a simple arithmetic mean was calculated for each of the 10 sensors from these 30 s. Additionally, the mean of all 10 sensors for each measurement position was calculated. By evaluating not the absolute values of sensor data but only the changes in inclination values concerning the 0° position, a maximum achievable accuracy of the sensor module of 0.006° under laboratory conditions was obtained, confirming the results of previous studies [43].

### 2.1. Further Preliminary Investigations

#### 2.1.1. Optimal Number of Sensors

**Figure 4:**
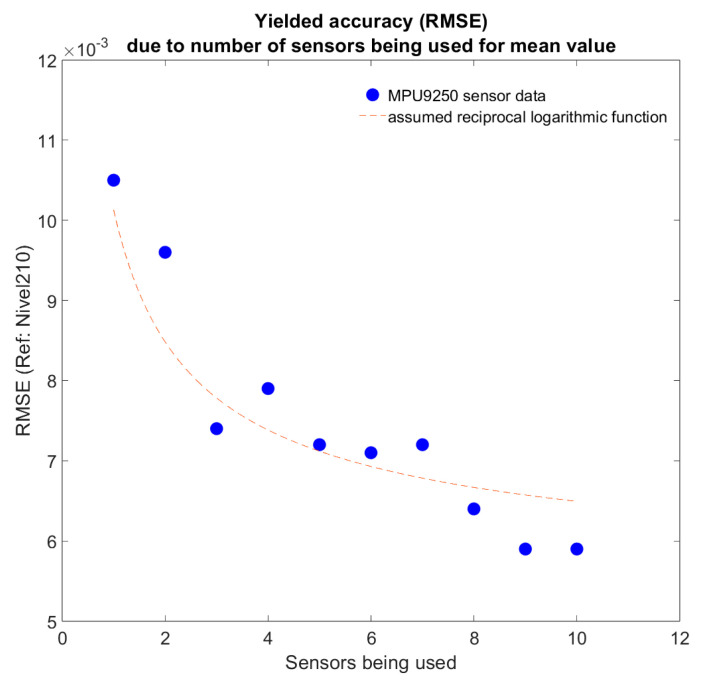
RMSE of mean sensor data vs. number of sensors being used.

The studies also clearly showed that, as expected, the accuracy increases with a larger number of sensors used for averaging. The extent of the accuracy gain was quantified, assuming an inverse-logarithmic relationship, as shown in Figure 4:

The results indicate that with four sensors, a good balance between sufficient accuracy and economic efficiency is achieved.

#### 2.1.2. Field Test on a Bridge Structure

**Figure 5:**
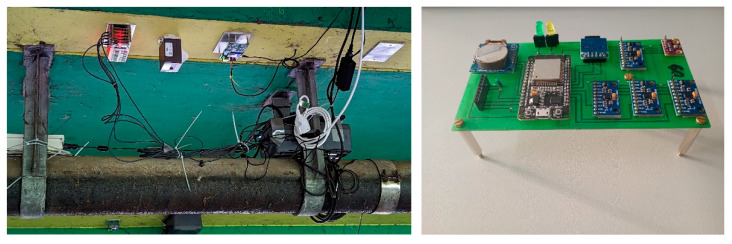
In total, 50 MEMS accelerometers on five PCBs stacked over each other (**left**) and one PCB with 4 MEMS accelerometers (**right**).

A second module (Figure 5 right), consisting of 4 MEMS accelerometers and an external temperature sensor connected to a microcontroller, was therefore created.

This module, alongside five stacked 10-sensor PCBs, was mounted on the underside of the Benediktus Bridge in Düsseldorf to collect long-term field data.

**Figure 6:**
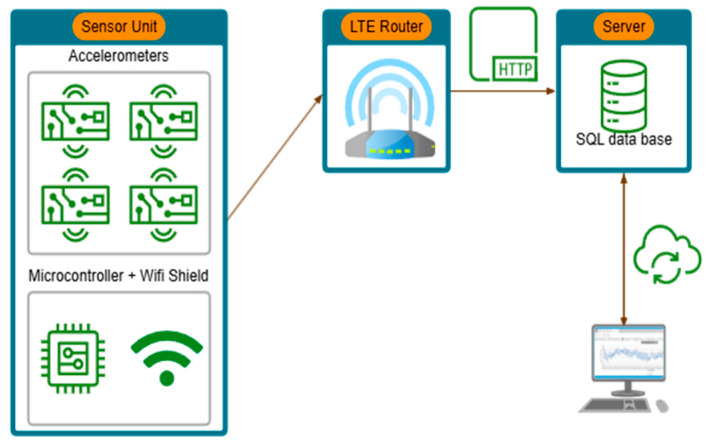
Schematic representation of data processing and storage chain.

To implement the typical data availability for geomonitoring, a structure was set up in which recorded data are transmitted by the microcontroller via WLAN and an LTE router to a web server and then stored in an SQL database using a PHP script (see Figure 6). This allows data not only to be read directly from the microcontroller but also to be retrieved online and live at any time.

The described laboratory and field tests demonstrate the fundamental usability of low-cost MEMS accelerometers in the context of structural monitoring. However, random and various systematic influences lead to measurement deviations. These are further quantified below, and appropriate measures are proposed.

### 2.2. Influence of Error Characteristics and Environmental Conditions on the Application Case

The error behaviour of sensors and the possibilities for modelling sensor behaviour have been presented in several publications, see, for example, [24,47,48]. Basically, error influences in MEMS inertial measurement units, as in all sensors, are divided into random and systematic measurement deviations. Random deviations are stochastically modelled and include white and coloured noise, which differ in intensity across the frequency spectrum. Systematic deviations can be further described as follows:

- Bias;
- Scale error;
- Non-orthogonality;
- Temperature-induced errors;
- Deviations due to ageing, mechanical stress, and other rather minor causes such as effects related to the electrical circuitry of the sensor chip.

**Figure 7:**
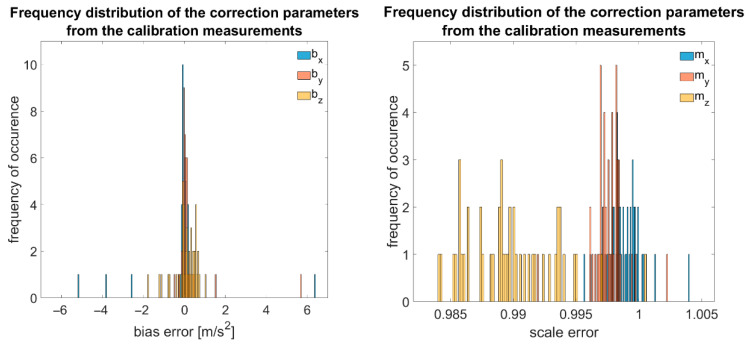
Frequency distribution of bias and scale factors of 50 MEMS accelerometers.

In the following, an estimation of the main errors and resulting measurement deviations will be provided. In an experiment, the calibration parameters of 50 MPU-9250 sensors were determined through the calibration procedure according to [48], followed by an equalisation calculation, and the frequency distribution was analysed. Results are summarised in Figure 7:

It can be seen that the bias in most cases lies within the range of ±0.7 m/s^2^ (σ≈1 m/s^2^, with the mean of all bias factors being 0.057 m/s^2^). The scale factors in our investigations range from 0.985 to 1, with non-orthogonalities at ±2% according to the datasheet. Additionally, there is nearly normally distributed statistical noise around the expected value, which, according to our investigations, is ≈460 μg/√Hz for the x- and y-axes and ≈800 μg/√Hz for the z-axis.

Furthermore, the influence of temperature on measurement results was examined: various MPU-9250 sensors were positioned at rest in a climate chamber, and changes in acceleration measurements were recorded over a temperature range from −20 °C to 50 °C.

**Figure 8:**
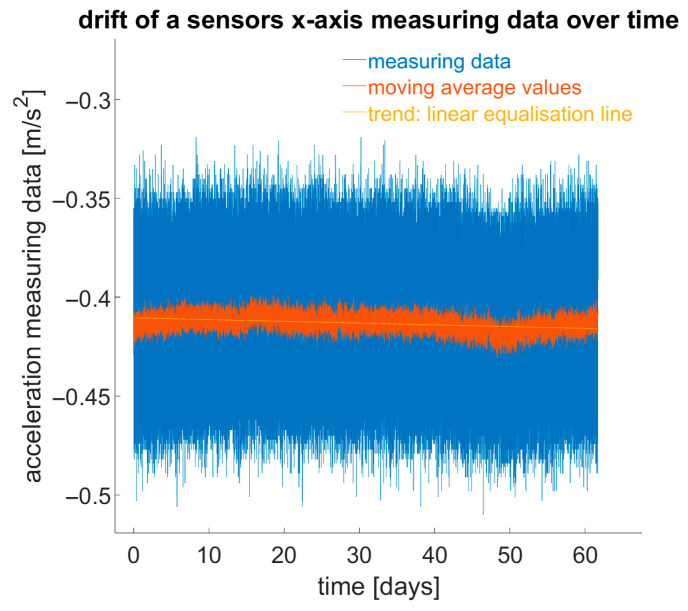
Drift of sensor data over time.

In another experiment, the temporally unstable drift behaviour, i.e., the slow, seemingly random change in measurement values over time, was observed and documented. For two months, 10 sensors were monitored at a constant temperature at rest to see how measurement values changed over time (Figure 8).

For the x- and y-axes, extrapolation over a year resulted in changes of up to 0.04 m/(s^2^·a), and for the z-axis, up to 0.1 m/(s^2^·a). It should be noted that this drift is random, and thus extrapolations have limited significance.

#### Estimation of Error Magnitudes and Comparison

When comparing all influences and determining the resulting error in inclination calculation, it becomes clear that the systematic temperature influence on measurement results is significant, as illustrated in the following list (data on respective error influences were taken from the sensors datasheet):

- **Bias:** reduced, as only changes in inclinations are considered for further analysis in monitoring.
- **Scale error:** max. 0.995 for x- and y-axes.

(6) $$r_{s}=\arctan\frac{{6.95\frac{m}{s^{2}}}^{*}}{6.93\frac{m}{s^{2}}}-\arctan\frac{0.995\cdot {6.95\frac{m}{s^{2}}}^{*}}{6.93\frac{m}{s^{2}}}\approx 0.14{}^{\circ}$$

- Orthogonality error: ±2%

(7) $$r_{o}=arctan\frac{{0\frac{m}{s^{2}}}^{**}}{9.81\frac{m}{s^{2}}}-arctan\frac{{0\frac{m}{s^{2}}}^{**}-0.02\cdot 9.81\frac{m}{s^{2}}}{9.81\frac{m}{s^{2}}}\approx 1.146{}^{\circ}$$

- **Noise:** minimised in the specific application through averaging.
- **Temperature dependency**: max. 0.4 m/s^2^ (according to datasheet and own analysis).

(8) $$r_{T}=arctan\frac{0.4\frac{m}{s^{2}}}{9.81\frac{m}{s^{2}}}=2.33{}^{\circ}$$

- **Temporal sensor drift:** x- and y-axes: up to $0.04\frac{m}{s^{2}\cdot a}$, z-axes: up to $0.1\frac{m}{s^{2}\cdot a}$.

(9) $$r_{t}=arctan\frac{0.04\frac{m}{s^{2}}}{\sqrt{{\left(0.04\frac{m}{s^{2}}\right)}^{2}+{\left(9.81\frac{m}{s^{2}}-0.1\frac{m}{s^{2}}\right)}^{2}}}=0.236{}^{\circ}/a$$

Assumptions for estimates: inclination occurs only on one axis.

* The maximum scale error is expected at a 45° inclination angle. This corresponds to a sensor acceleration value of $a_{x}=6.95\frac{m}{s^{2}}$ and $a_{z}=6.93\frac{m}{s^{2}}$ given a scale error of 0.995.

** The maximum orthogonality error is expected at a 0° inclination angle. This corresponds to acceleration values of the sensor’s orthogonal axes of 0 m/s^2^ and 9.81 m/s^2^.

As the calculations show, this is an estimate of expected maximum values for each “error category”. This estimation does not reflect a specific laboratory or field test, but rather indicates that temperature influence significantly exceeds other factors. Additionally, the assumption in our application of monitoring infrastructure is that the MEMS sensor should only detect changes in inclinations. These inclination changes are small and it is assumed that the systematic deviations/errors due to bias, scale, and orthogonality remain nearly constant with small inclination changes and are largely eliminated by relative comparison to a zero measurement (inclination change). Particularly noteworthy are the non-orthogonalities of axes among themselves due to larger production tolerances in the low-cost sector, which are eliminated to a great extent from fewer degrees of inclination changes by difference formation in the mentioned application field.

Temporal drift of measurements, usually expressed as Bias Instability and Rate Random Walk, cannot be assumed constant as it is assumed to be a stochastic process and often changes direction. Although usually modelled as a stochastic process [49], Bias Instability and Rate Random Walk contain deterministic components such as ageing or thermal and mechanical effects. This influence can cause significant measurement deviations over time. Due to its non-systematic nature, compensating for or modelling this effect is difficult [26]. This topic will not be discussed further in this paper. The following part of this publication addresses the most significant influence on the accuracy of measurement results: the impact of temperature and possible measures to minimise it.

### 2.3. Temperature Effect Compensation

The temperature dependence of measurements from MEMS accelerometers primarily results from changes in the mechanical properties of the sensor’s micro-components due to temperature fluctuations [50]. Numerous studies have addressed this, some with notable results attempting to achieve stability in temperature behaviour through targeted mechanical and/or electrical optimisations; see, for example, [51,52,53]. While these approaches may significantly improve the temperature resistance of MEMS accelerometers, they also lead to increased complexity of the components, thus raising manufacturing effort and production costs. This paper does not further elaborate on these approaches; instead, it focuses on the analysis of the capabilities of low-cost MEMS accelerometers. To remain within the scope of low effort and cost, components commonly used in consumer electronics are adapted with minimal alteration for geomonitoring applications.

#### Experiment Setup and Execution

**Figure 9:**
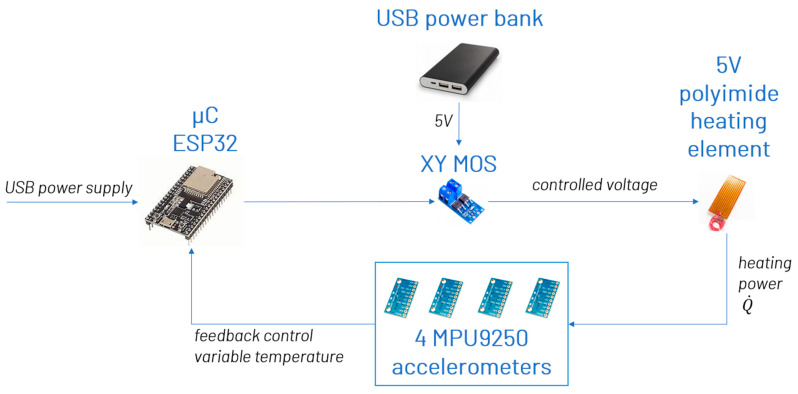
Schematic representation of system configuration.

A cost-efficient approach is presented below, wherein the principal system setup from Figure 6 is expanded with sensor heating. MEMS accelerometers were incorporated into a temperature control loop, with the sensor’s internal temperature serving as the control variable. On the hardware side, the board was upgraded with a MOSFET driver (XY MOS), actuated by a PWM signal from the microcontroller. This driver controlled a 5 V polyimide heating element in response to the PWM signal, powered by a constant 5 V supply (a USB power bank), see Figure 9.

The heating element was secured to the four sensors, and using thermal paste ensured good thermal contact between the heater and sensors. The sensor temperature, acting as the control variable, was fed into the microcontroller, guiding the actuator, i.e., the MOSFET driver with the attached heating element, within the temperature control loop. A target temperature of 67 °C was set to provide a buffer against maximum expected ambient temperatures, ensuring that this target, even without heating, is not exceeded due to direct sunlight or other factors. This method obviates the need for cooling, which would necessitate more complex hardware solutions.

**Figure 10:**
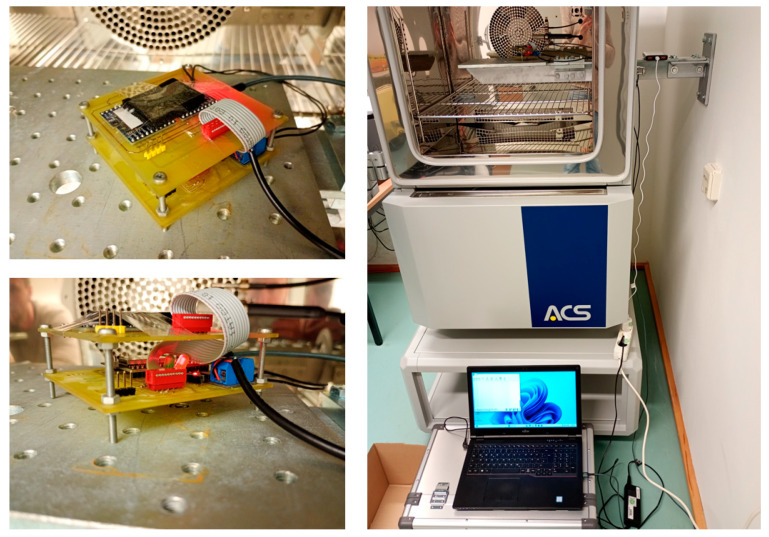
Experimental setup in a climate chamber: four MPU-9250 sensors on the top-layer PCB, connected with a flat cable to the processing unit at the bottom layer.

This setup was installed in a climate chamber simulating a (extreme) daily temperature cycle from −20 °C to +50 °C over 14 days, covering the temperature extremes likely to be encountered in moderate latitudes, considering even direct sunlight. This cycle was replicated over 24 h periods for 14 days (resulting in 14 temperature peaks and 14 troughs). The temperature control was engaged for the first seven days to maintain stable sensor temperatures, and switched off during the subsequent seven days to compare results and quantify the efficacy of the measure. Figure 10 shows the experimental setup in the climate chamber.

It is essential to differentiate between sensor temperatures and climate chamber temperatures. The climate chamber temperature approximates the sensor’s real environmental temperature, while the sensor’s temperature in the MPU-9250 is elevated due to operational processes and resultant heat loss. Depending on the individual sensor, this temperature can be up to 20 °C higher than its actual ambient temperature.

## 3. Results

The evaluation of temperature effect compensation in low-cost MEMS accelerometers is divided into two parts: a hardware-based measure involving electrical heating of the sensors and a post-processing measure compensating for non-idealities in heat transfer. Despite the temperature control, factual fluctuations in sensor temperatures occurred, attributed to non-ideal heat transfer.

### 3.1. Electrical Heating of Sensors

According to eq. 1.1, inclination angles (relative to the geoid) can be determined using acceleration data due to gravity acting in the direction of the natural vertical. In the described setup, aside from a constant offset to the horizontal plane, these inclination angles are mainly affected by temperature-induced errors. As the sensors are at rest, observed changes in inclination can be attributed to temperature variations. It will become evident that these temperature errors in this case can primarily be traced to two causes: internal temperature effects within the sensor, as derived by, e.g., [24], and elastic deformations of the sensor’s substrate.

A moving average over 1500 values (about 10 min) was applied to all the datasets to reduce noise and highlight physical processes. While this is a relatively long averaging period, it is justified by the enhanced insights gained as well as the slow processes that are the focus of this study in structural monitoring.

**Figure 11:**
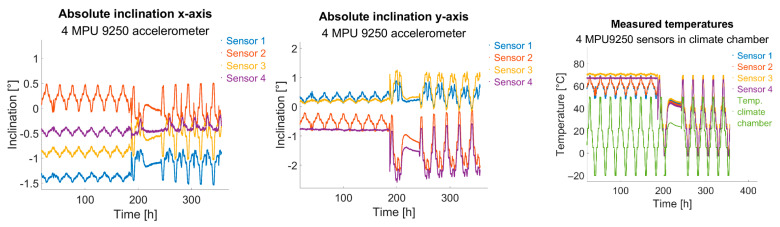
Left and centre: inclination changes due to temperature variation while sensors are being static at rest // Right: sensor temperatures and climate chamber temperature.Interestingly, despite sensor 4 maintaining almost ideal constant temperature due to temperature control (see right plot in Figure 11 in purple), its x-axis inclination values still exhibited changes (see left plot in Figure 11 and Figure 12). With the sensor assumed to be at rest and at a constant temperature, such variation is not expected. Conversely, y-axis inclination remained nearly stable, apart from slight temporal drift during the trial; see centre plot in Figure 11. Given the correlation between x-axis pseudo-inclination change and temperature (correlation factor −0.93), it is likely that thermal differences within the board (up to 90 °C between sensor and surroundings) induced mechanical stresses, causing real inclination via thermal strains—possibly a single-axis board warp from these stresses.

In Figure 11, it is apparent that between 180 h and 230 h, the temperature control was deactivated, allowing the chamber to warm to room temperature. During periods when the sensors were heated, pseudo-inclination changes were significantly reduced. The sensor temperature of sensor 4 (purple) served as the control variable for the temperature loop, hence showing the lowest inclination change. Discrepancies among the sensors can be attributed to non-ideal heat transfer from heater to sensor: despite using thermal paste, the heating power could not be perfectly uniformly distributed to all sensors, creating varied internal temperature trends.

**Figure 12:**
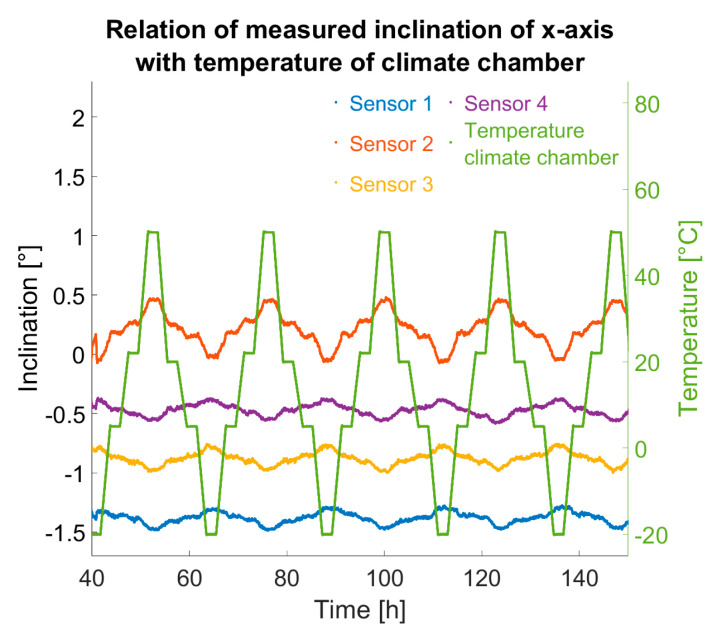
Relation of calculated inclination of sensors at rest with temperature variations in climate chamber at temperature-controlled phase of experiment.

### 3.2. Linear Correlations Due to Temperature Control

**Figure 13:**
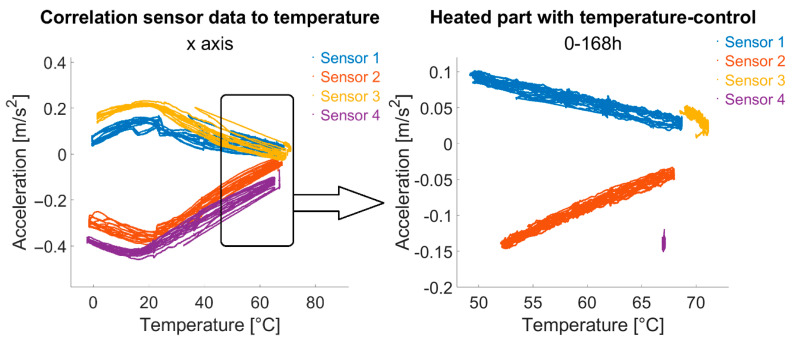
Acceleration vs. sensor temperature for x-axis (moving average values); **left**: 14 days data; **right**: only first 7 days with temperature control.

The effect of temperature on sensors is most evident in an a-T (acceleration vs. temperature) diagram, contrasting raw accelerometer readings to temperature data (Figure 13).

In the left plot, for example, the x-axis acceleration is plotted against temperature, showing a clear correlation between the two parameters. In addition, a sign reversal of the correlation behaviour can be observed—for all sensors—in the range of approx. 20 °C sensor temperature (approx. 5 °C ambient temperature). A linearity of the correlation can be assumed both in higher temperature ranges and in lower temperature ranges below, but not over the entire temperature range that occurs in the reality of building monitoring. On the left, only data from the first seven temperature-controlled days is shown, clearly highlighting linearity. The purple cluster represents sensor 4 data, with nearly no change in temperature and acceleration due to ideal temperature regulation.

### 3.3. Data Analysis and Processing

**Figure 14:**
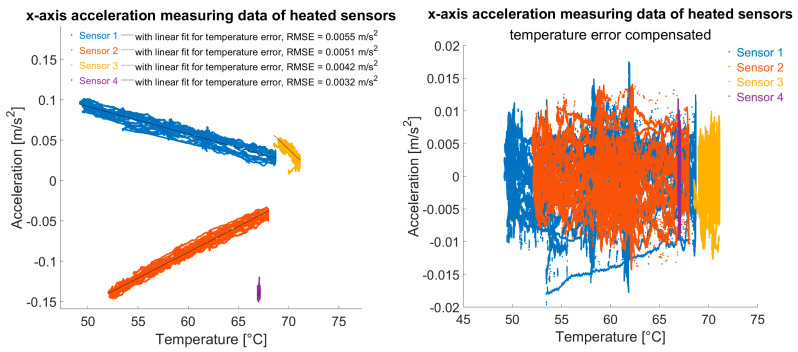
The x-axis acceleration measuring data of temperature-controlled sensors; (**left**): with least squares linear fit as an approximation for the temperature error; (**right**): temperature compensated.

When heated, sensors had largely temperature-error-free readings. Heating systematically stabilised temperature influence. Even if the control loop shows ±5 °C variations, local temperature compensation via linear approximation can improve reducing temperature-related errors (Figure 14).

Such a linear compensation, but without a control loop, would not be promising due to correlation sign reversals as pointed out in the left plot of Figure 13. However, compensation of the measurement data in the small temperature range, e.g., due to a temperature control loop mentioned above, could be carried out “live” in the sensor or, as in the proposed monitoring system in Figure 6, on the server using pre-defined correction functions.

#### 3.3.1. Simple Arithmetic Mean of MEMS Sensor Data

**Figure 15:**
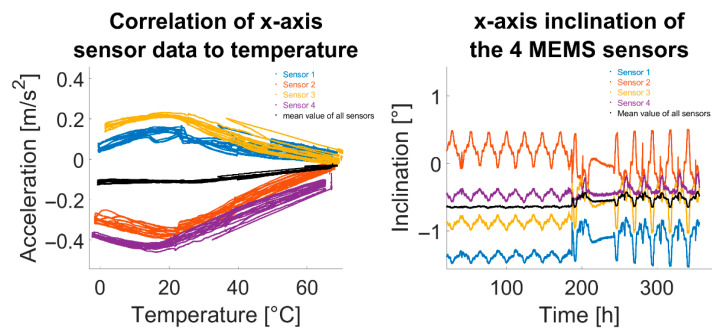
Examples of improved acceleration and inclination data by forming the arithmetic mean of all sensors.

An additional approach is averaging several sensor data. Figure 15 shows how averaging across all sensor data from the climate chamber test enhanced system performance. Using more sensors increases the chance that temperature-correlation effects will offset each other.

#### 3.3.2. Heating and Cooling Phases

**Figure 16:**
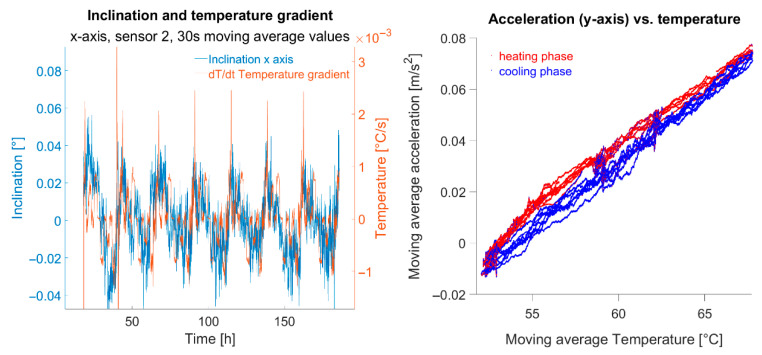
(**left**): relation between temperature gradient and sensor output (or resulting inclination, respectively); (**right**): heating phase (red) and cooling phase (blue) of the temperature-controlled part of sensor 2.

Eliminating temperature effects should leave no systematic influences due to temperature in cleansed data. On the left of Figure 16 x-axis inclinations during the heated test phase are compared against corresponding temperature gradients. Apparently residual systematic effects remain in reduced data. It is evident that a decline or rise in inclination corresponds with negative or positive temperature gradients, respectively, indicating different sensor behaviour during warming versus cooling.

The right plot in Figure 16 shows an example of the a-T plot of the temperature-controlled part of the experiment for the y-axis values of sensor 2. Cooling processes are shown in blue, heating processes in red. It can be seen that heating processes take the “upper path” while cooling processes take the “lower path”. The physical cause of this phenomenon is assumed to be internal to the sensor. To improve accuracy, second-order polynomial approximations for warming and cooling phases were applied separately. Despite being detectable, this effect, using separate temperature error functions for heating and cooling, did not enhance accuracy parameters. Further data processing, such as higher-order polynomial approximation or applying a simple arithmetic mean of, e.g., 30 s on the data, did not yield significant improvements.

### 3.4. Summary of Result Data

Table 1 compares all described measures for improving MEMS accelerometer accuracy. Inclination data (x- and y-axes) are provided as standard deviation (RMSE, 1σ) and maximum inclination error (“max. IE,” i.e., span of measurement variability). Factor k, a quotient of accuracy improvement compared to raw data without intervention (A), provides a measure of the increase in accuracy. Improvements included implementing the temperature regulation loop (B), followed by compensating the remaining temperature effects using a linear error approximation (C), and a polynomial second-degree approximation for temperature error function (D). Lastly, separate heating/cooling phase approximations were conducted (E).

Root mean square error (RMSE) pertains to differences between measured relative inclination and factual rest position at zero inclination. According to Equation (5),

(10) $$RMSE=\sqrt{\frac{1}{n}\cdot \sum_{i=1}^{n}{\left({\bar{r}}_{m,rel}-{\bar{r}}_{ref}\right)}^{2}}$$

Here, ${\bar{r}}_{ref}=0$ is the reference, the vector of true values, and ${\bar{r}}_{m,rel}$ is the measured, relative inclination as the vector with length n of all measured inclination values. This is reduced by the offset $r_{0}$, caused by the sensor bias.

(11) $${\bar{r}}_{m,rel}={\bar{r}}_{m}-r_{0}={\bar{r}}_{m}-\frac{1}{i^{48h}}\cdot \sum_{i=1}^{i^{48h}}r_{m,i}$$

The offset is derived from averaging all inclination values for the first two days. If, for example, the first measured value rather than the mean value of the first two days was used to form relative values, the calculation of the RMSE would be distorted depending on whether the first measured value was close to a maximum or minimum of the time series. In addition, averaging is limited to the first 24 h or 48 h, so that the effect of the long-term bias drift of the sensors is not yet measurable.

#### Discussion of Results

The results in the table illustrate what was already recognisable from the figures shown: heating the sensors to a stable temperature using the temperature control and the subsequent linear compensation of remaining residual errors significantly improve the quality of the measurement data. The accuracy (1σ) of the relative inclinations reaches 0.015–0.04° for the individual sensors, and even 0.006° when using the mean value from several sensors (max. IE: 0.1–0.2° for the individual sensors or 0.04–0.06° as the mean value from the four sensors). The improvement factor k, which measures the quality of the respective measures, reaches a factor of 16 (max. IE) or 33 (1σ) with very good temperature control (sensor 4). Further measures, such as the formation of simple arithmetic mean values over the time of the measurement process, the use of higher order polynomials in the approximation of the temperature error function, or the separate approximation of heating and cooling phases of the sensors, do not bring any further significant improvements. The results obtained from the mean values of several sensors would thus even fulfil the accuracy requirement mentioned at the beginning of the concrete example of the Benediktus Bridge in Düsseldorf, in order to be able to detect static deflections of 2 mm in a cantilever with a length of 3.24 m of a box girder bridge.

Elastic deformation of the printed circuit boards on which the accelerometers were soldered, caused by mechanical stress due to large thermal differences in the immediate vicinity of the sensors, is thought to be the reason for the measured inclinations around the x-axis in the rest position, which were still visible in sensor 4 despite almost ideal temperature control. It is planned to investigate the extent to which stable temperature control of the entire enclosure can eliminate this effect. It has been shown that good temperature control (max. +/−0.2° deviation of the controlled variable from the target variable) gives approximately the same good results in terms of achievable accuracy as the combined solution of poor-quality temperature control (e.g., max. +/−10° deviation of the controlled variable from the target variable) and subsequent temperature compensation. If precise and extremely stable temperature control is not possible for design, economic, or other reasons, such as extreme ambient temperature conditions, a combined solution of a low-quality temperature control loop and temperature error compensation provides a comparably good solution.

Pure compensation using a temperature correction function without heating is not recommended as the correlation behaviour of a sensor between temperature and acceleration is more difficult to approximate over a wide temperature range. For the sensors investigated, the correlation behaviour leaves the linear relationship in the lower temperature range and then reverses the sign. Without prior laboratory testing, this effect would lead to larger approximation errors.

## 4. Summary and Outlook

This study presents the methodology and test results of the development of low-cost MEMS inclination sensors for infrastructure monitoring. Proposed improvements were explained and their impact on the accuracy of the overall system was quantified. Simulated tests in a climate chamber, in which four low-cost MEMS accelerometers were exposed to a daily temperature range of −20–+50 °C for 14 days, showed that an extension of the sensor platform by a simple temperature control loop with a polyimide heating element to achieve a stable sensor temperature was sufficient to increase the accuracy of the system by a factor of 16 (max. inclination error IE) or 33 (1σ accuracy), respectively. When the values of several sensors were combined into an overall mean value, the accuracy (1σ) of the relative inclinations reached 0.006° (for max. IE: 0.04–0.06°). This shows that the laboratory value for the accuracy of the measurement system mentioned at the beginning, which was determined without the influence of temperature, can now be achieved despite the influence of temperature due to the various measures, thereby largely eliminating the temperature dependency of the sensors. With the sensor module presented, structural monitoring and SHM concepts can therefore be implemented in accordance with the accuracies achieved here, as shown, for example, by the tolerance limits and derived accuracy requirements of the practical example of the Benediktus Bridge in Düsseldorf. Validation under field conditions is therefore the next logical step that should be taken.

The cost of adding the temperature control loop to the measurement system was EUR 3–5 (polyimide heater and MOSFET driver (XY MOS) to control the heater). The other components of the measurement system, consisting of four MPU-9250 sensors and an ESP32 microcontroller, cost around EUR 60.

If good temperature control (max. +/−0.2° deviation of the controlled variable from the setpoint) can be achieved, this is by far the most important measure for improving accuracy. If this quality of control is not possible for design or economic reasons, a combination of a lower quality temperature control loop (e.g., max. +/−10 °C deviation of the controlled variable from the setpoint) with the identification of a linear temperature error function and subsequent compensation for temperature effects is recommended. The resulting improvements are comparable to pure, accurate temperature control. The necessary identification of the linear relationship between changes in temperature and acceleration could even be carried out on site at the object. The prerequisites for this are that the sensors are exposed to a representative temperature range for the duration of the identification process, that the structure is not exposed to any long-term geometric changes during the investigation period, and finally that the identification process is sufficiently short so that long-term bias drift effects of the sensor do not falsify the parameter estimation. The relationship or correlation between temperature and acceleration can now be derived from the data recorded during the investigation period and applied to future measurement data for the purpose of temperature effect compensation without the need for complex laboratory testing. The extent to which this procedure ensures the subsequent quality of measurement data compared to laboratory-based identification of the temperature-acceleration behaviour of a sensor remains to be investigated. With the presented setup with MPU9250 MEMS sensors, the target temperature for a poor-quality temperature control loop, with temperature variations of a few degrees Celsius, should be at least 15 °C so that the sensor temperature does not reach a range where the linear relationship in the temperature-acceleration correlation behaviour is lost.

In addition, the tests on one of the two tilt axes showed changes in tilt values with temperature, even with almost perfectly temperature stable control of a sensor in the rest position. These changes are attributed to thermally induced mechanical stresses that lead to elastic deformation of the test structure, which consists of four accelerometers soldered to a printed circuit board with conductive tracks. The verification and elimination of this effect by means of a sensor module, in which not only the individual sensors themselves but also the entire housing is temperature-stably regulated, is the subject of further investigations.

## Figures and Tables

**Table 1:** Comparison of results in °, S is for the respective sensor, x and y are for both axes, and ∅ is for the mean value of all sensors.

		**RMSE (1** **σ** **)**					**Maximum Inclination Error**				
		**S 1**	**S 2**	**S 3**	**S 4**	∅	**S 1**	**S 2**	**S 3**	**S 4**	∅
A	x	0.249	0.259	0.282	0.086	0.093	0.739	0.937	0.809	0.469	0.253
	y	0.177	0.763	0.402	0.773	0.244	0.981	2.156	1.418	2.008	0.547
B	x	0.048	0.143	0.057	0.050	0.008	0.213	0.568	0.246	0.217	0.045
	k_x_	5.178	1.810	4.988	1.706	11.936	3.475	1.640	3.286	2.164	5.616
	y	0.099	0.162	0.043	0.024	0.009	0.431	0.638	0.246	0.156	0.067
	k_y_	1.779	4.714	9.412	32.608	28.080	2.278	3.370	5.775	12.863	8.153
C	x	0.015	0.021	0.017	0.017	0.006	0.105	0.130	0.127	0.114	0.042
	k_x_	17.702	12.407	18.418	5.850	16.333	7.016	7.201	6.365	4.113	6.017
	y	0.038	0.039	0.028	0.023	0.008	0.195	0.165	0.144	0.128	0.059
	k_y_	4.657	20.461	15.399	33.310	33.931	5.037	13.108	9.823	15.724	9.289
D	x	0.014	0.021	0.016	0.015	0.006	0.102	0.124	0.106	0.112	0.038
	k_x_	17.702	12.231	18.064	5.584	16.333	7.257	7.555	7.616	4.183	6.598
	y	0.038	0.037	0.026	0.023	0.007	0.194	0.149	0.130	0.125	0.055
	k_y_	4.633	20.461	15.399	33.167	33.014	5.053	14.481	10.886	16.038	9.911
E	x	0.013	0.018	0.016	0.015	0.006	0.105	0.144	0.107	0. 112	0.040
	k_x_	18.909	14.817	18.064	5.584	16.927	7.040	6.488	7.552	4.183	6.255
	y	0.035	0. 036	0.026	0.023	0.007	0.217	0.166	0.132	0. 125	0.054
	k_y_	5.087	21.438	15.399	33.167	34.900	4.528	13.013	10.754	16.050	10.057

## Data Availability

The original contributions presented in this study are included in the article. Further inquiries can be directed to the corresponding authors.
